# P102 Influence of extracellular zinc on MBL activity and carbapenem susceptibility

**DOI:** 10.1093/jacamr/dlaf230.109

**Published:** 2025-12-04

**Authors:** Anurag Payasi, Shailesh Kumar, Saransh Chaudhary, Anmol Aggarwal

**Affiliations:** Venus Medicine Research Centre, India; Venus Medicine Research Centre, India; Venus Medicine Research Centre, India; Venus Medicine Research Centre, India

## Abstract

**Background:**

MBLs require zinc ions (Zn²⁺) as essential cofactors for their activity. Previous research has shown that physiological zinc concentrations in human plasma range from approximately 0.8 to 1.2 μg/mL. This study investigated how variations in extracellular zinc concentration within standard culture media impact MBL activity and the resulting MICs of carbapenems.

**Methods:**

The *in vitro* susceptibility of clinical MBL-producing *Escherichia coli* and *Klebsiella pneumoniae* isolates (NDM-1, VIM-1, combinations; *n*=10), ESBL controls (*E. coli* ATCC 35218 (TEM-1), *K. pneumoniae* ATCC 700603 (SHV-18)), and a WT (*E. coli* ATCC 25922) were evaluated. Zinc-depleted CAMHB was prepared using Chelex-100 resin, and zinc levels were quantified by ICP-MS. Following depletion, the media were reconstituted with standard levels of calcium, magnesium and iron. MICs for carbapenems (meropenem, imipenem, doripenem, ertapenem) and control antibiotics (amikacin, polymyxin B) were determined by broth microdilution (ISO 20776-1) in three parallel media: (i) untreated CAMHB, (ii) zinc-depleted CAMHB and (iii) zinc-depleted CAMHB supplemented with 5 μM Zn²⁺.

**Results:**

ICP-MS analysis confirmed that Chelex treatment effectively reduced the intrinsic zinc concentration in CAMHB by over 63% (from 0.964 μg/mL to 0.352 μg/mL). In this zinc-depleted medium, MBL-producing isolates exhibited a significant 2- to 128-fold reduction in carbapenem MICs compared to untreated CAMHB. Crucially, supplementing the zinc-depleted broth with 5 μM Zn²⁺ (1.43 μg/mL) restored carbapenem MICs to levels comparable to, or marginally exceeding, those observed in untreated broth. In contrast, the MICs for MBL-negative isolates (ESBL-producers and WT) and for non-β-lactam antibiotics remained unchanged across all media conditions, confirming the effect was specific to MBL activity. Higher zinc supplementation (>5 mM) yielded no further significant increases in MICs.

**Conclusions:**

Extracellular zinc availability is a critical determinant of MBL-mediated carbapenem resistance *in vitro*. Standard susceptibility testing media may contain supra-physiological concentrations of zinc, resulting in a higher number of false negative susceptibility results in routine clinical testing. Failure to control for zinc can lead to discrepancies in MIC values and misinterpretation of resistance profiles. These findings emphasize the need to standardize zinc concentrations in diagnostic media and to further investigate the clinical relevance of zinc availability at the site of infection.

